# Progestin Receptor-Mediated Reduction of Anxiety-Like Behavior in Male Rats

**DOI:** 10.1371/journal.pone.0003606

**Published:** 2008-11-05

**Authors:** Catherine J. Auger, Robin M. Forbes-Lorman

**Affiliations:** Department of Psychology, University of Wisconsin, Madison, Wisconsin, United States of America; Pennsylvania State University, United States of America

## Abstract

**Background:**

It is well known progesterone can have anxiolytic-like effects in animals in a number of different behavioral testing paradigms. Although progesterone is known to influence physiology and behavior by binding to classical intracellular progestin receptors, progesterone's anxiety reducing effects have solely been attributed to its rapid non-genomic effects at the GABA_A_ receptor. This modulation occurs following the bioconversion of progesterone to allopregnanolone. Seemingly paradoxical results from some studies suggested that the function of progesterone to reduce anxiety-like behavior may not be entirely clear; therefore, we hypothesized that progesterone might also act upon progestin receptors to regulate anxiety.

**Methodology/Principal Findings:**

To test this, we examined the anxiolytic-like effects of progesterone in male rats using the elevated plus maze, a validated test of anxiety, and the light/dark chamber in the presence or absence of a progestin receptor antagonist, RU 486. Here we present evidence suggesting that the anxiolytic-like effects of progesterone in male rats can be mediated, in part, by progestin receptors, as these effects are blocked by prior treatment with a progestin receptor antagonist.

**Conclusion/Significance:**

This indicates that progesterone can act upon progestin receptors to regulate anxiety-like behavior in the male rat brain.

## Introduction

Progesterone (P) can have anxiolytic-like effects in animals in a number of behavioral paradigms [1]–[6]. P is known to influence physiology and behavior by binding to classical intracellular progestin receptors (PR), which in turn can act at the genome to regulate gene expression [7]–[9]. The anxiolytic-like actions of P are believed to occur mainly following its conversion by 5α-reductase to one of its ring A reduced metabolites, 3α-hydroxy-5α-pregnan-20-one (allopregnanolone) [10], [11]. The effects of allopregnanolone occur in a rapid non-genomic membrane-mediated manner [11], [12], as it allosterically modulates GABA_A_ receptors [13], [14], and it is through this interaction that allopregnanolone has its anxiety-reducing effects on behavior [2], [15]–[17].

Different approaches have been used to examine the relative importance of P versus allopregnanolone in reducing anxiety-like behavior. Some of these studies have uncovered results inconsistent with the contention that there is a single pathway by which anxiety is reduced. While one study utilizing female progestin receptor knockout (PRKO) mice did report anxiolytic-like effects of P [18], another study reported that male PRKO mice displayed more anxiety-like behavior in the elevated plus maze. These animals spent significantly less time in the open arms compared to their wild type background strain [19]. These data suggest that PR may play some role in regulating anxiety-like behaviors in males. If reduction in anxiety-like behavior is only accomplished by neurosteroid action in the brain, then mice containing the PRKO mutation should not have altered anxiety levels. On the contrary, it appears that the absence of PR in these mice leads to more anxiety-like behavior compared to their wild type controls, suggesting that PR might be involved in reducing anxiety-like behavior.

Further support comes from studies that block 5α-reductase. This treatment interferes with the production of allopregnanolone. Mice deficient in this enzyme still showed anxiolytic-like behavior in response to P within the elevated plus maze, suggesting that P was still reducing anxiety-like behavior through some mechanism other than its conversion into allopregnanolone [20]. Similarly, lactating females, that have endogenously high levels of P, still showed less anxiety-related behavior than control rats even after treatment with an inhibitor of 5α-reductase, [21]. In this experiment, however, the 5α-reductase inhibitor was effective, as the turnover of P to allopregnanolone was significantly reduced. Still anxiety-related behavior was low. These data suggest that an additional pathway to regulate anxiety may exist, a pathway which does not necessitate the conversion of P to its neuroactive metabolites, and in which PR appear to play some role in regulating anxiety-like behavior.

In the current study, we aimed to test the hypothesis that P can modulate anxiety-like behavior partly by acting upon PR in the male brain. Therefore, we examined the anxiolytic-like effect of P in the presence or absence of the PR antagonist RU-486 using the elevated plus maze, a validated test of anxiety [22], [23], and the light/dark chamber. We present evidence here to suggest that, in male rats, the anxiolytic-like effects of P are indeed mediated, in part, by its actions at the PR, as the anxiolytic-like actions of a physiologically-relevant dose of P can be blocked by a PR antagonist.

## Materials and Methods

This research was approved by the University of Wisconsin Animal Care and Use Committee.

### Animals

Adult male Sprague Dawley rats from our breeding colony (400–600 g; breeders from Charles River Labs, Inc., Wilmington, MA) were housed under a 12∶12 light/dark cycle (lights off at 1100 h) with food and water available ad lib. As previous studies have shown that progestogens can reduce endogenous levels of T by directly inhibiting testicular steroidogenesis [24]–[27],rats were castrated at approximately 3 months of age and implanted with s.c. Silastic® implants (2.5 cm long, 1.5 mm inner diameter, 2.4 mm outer diameter, Dow Corning Corp. Midland, MI) filled with testosterone (T).

### Elevated plus maze

The elevated plus maze, a validated and widely used test of anxiety in rodents [22], [23], consists of 2 opposing runways, one open and one closed, each measuring 100 cm in length and constructed of black Plexiglas. Each arm of the closed runway is fitted with 39 cm high Plexiglas walls on either side of the runways. The maze stands 50 cm off the floor. The rats were placed in the center of the maze, where the two arms intersect, facing an open arm. The animal's behavior in the maze was recorded for 5 minutes with a video camera connected to a DVD player for later behavioral observation. DVD recorded material was converted to MPEG format using VideoWave® Professional (Roxio, a division of Sonic Solutions, Novato CA) and then analyzed using The Observer® (Noldus Information Technologies, Wageningen, The Netherlands) by an experimenter blind to the treatment groups. Parameters quantified were the number of entries into the open and closed arms, and total time spent in the open and closed arms and the center chamber. An entry was counted when all four paws crossed into a certain portion of the maze.

### Light/dark chamber

The light/dark chamber consists of a polycarbonate chamber that is divided into one lighted chamber (39×35 cm) and one darkened chamber (39×25). A piece of polycarbonate made to be opaque is used to separate the light and dark sides of the chamber via insertion into specially crafted guides to ensure a tight fit across the entire width of the chamber. There is also an opening on the lower edge of this insert (5×10 cm) so the animal can move freely from one part of the chamber to the other. The dark side of the chamber is constructed of polycarbonate that is made to be opaque and this side of the chamber is equipped with an opaque lid. A white incandescent light is situated above the light side of the chamber. The walls of this side of the chamber are constructed of clear polycarbonate. The animal was placed in the middle of the light side of the chamber facing away from the opening toward the darkened side. A cross from one compartment to the other was recorded when all four paws were in one compartment. The animals were observed in the chamber for 5 minutes by an experimenter blind to the treatment groups. Parameters quantified were latency to cross over to the dark compartment of the chamber, and total amount of time spent on the lit side of the chamber.

### Data analysis

Statistical comparisons were carried out using Sigma Stat statistical software for Windows v3.11 (Systat Software, Inc., Point Richmond, CA). Data were compared with a One-Way ANOVA, and further analyzed using the Fisher LSD Method. The level of significance was set at p<0.05.

### Treatment

Six weeks following surgery rats received s.c. injections of either RU-486 (Sigma-Aldrich, St Louis, MO; 5 mg per animal) or vehicle once daily for 3 days. On each of these days, 2 hours following the RU-486 or vehicle injection animals received an injection of either P (Sigma-Aldrich, St Louis, MO; 1 mg per animal) or sesame oil. The dosage of RU-486 used was chosen because a dose response study [28] indicated that this dose was successful at blocking the effects of P on behavior in female rats and guinea pigs. Also, in a study that examined the anti-glucocordicoid properties of the same dose of RU-486 as has been used in the current experiment, cortisol treatment given simultaneously with RU-486 was not able to prevent RU-486 from blocking P-facilitated behavior [29]. These data suggest that this dose of RU-486 is blocking P-induced behavior by interfering with the activation of PRs. The dosage of P was chosen because it yields physiological levels of P that are observed following stress in male rats [30], [31], and is sufficient to induce sexual behavior in female rats [32], [33]. Four hours after the final P injection animals were tested in the elevated plus maze and light/dark chamber during the dark phase of the light cycle under dim red light.

### Enzyme Immunoassay (EIA)

At least 500 ul of blood was collected from treated animals and centrifuged at ∼9700 RPMs or 10,000 gs for 10 minutes. Next, serum was removed and stored in a clean tube at −20°C until used in T or P EIA (Cayman Chemical Company, Ann Arbor, MI). Both EIAs are based on the competition between the steroid of interest and steroid-acetylcholinesterase conjugate for a limited number of steroid specific binding sites. T or P standards were prepared according to the manufacturer's instructions. Following the preparation of standards, these and the serum samples from our treated animals were loaded into a 96-well plate, as well as the necessary controls. Next, the T- or P-acetylcholinesterase tracer was added to most of the wells followed by addition of the T or P antiserum to most of the same wells. The plate was then left to incubate for 1 hour for the P assay and 2 hours for the T assay at room temperature on an orbital shaker. After this incubation, the contents of the plate were discarded and the plate was rinsed 5 times with wash buffer supplied by the manufacturer. Following these rinses, Ellman's Reagent, the developing reagent supplied by the manufacturer, was added to the empty wells. The plate was then left to develop in the dark while placed on an orbital shaker. The developing process takes about 1 hour or until the absorbance of the maximum binding wells equal 0.3 A.U. Following the developing process, the plate is read at a wavelength between 405 and 420 nm with a plate reader. All samples for hormone measurement were quantified in the same assay. The assay specificity is 100% for T and P, respectively. The intra-assay coefficient for each assay was 9.1% for the T and 8.5% for the P assay, respectively. The detection limit of each assay was 6 pg/ml and 10 pg/ml for the T and P assay, respectively. Results were calculated using a computer spreadsheet program provided by Cayman Chemicals (www.caymanchem.com/eiatools/promo/kit).

## Results

### The effect of RU-486 on the anxiolytic-like effect of P in the elevated plus maze

**Figure 1:**
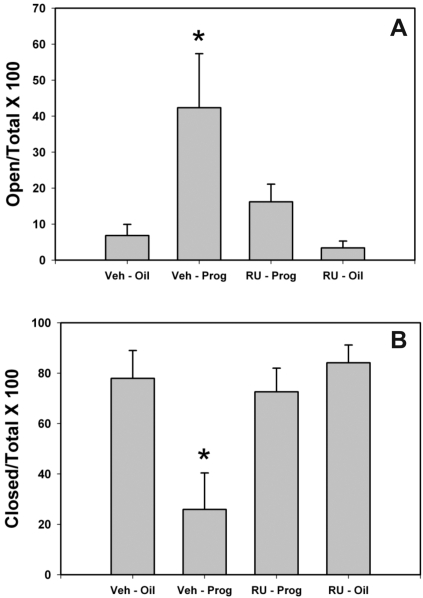
The effect of P and RU-486 on the percent time spent in the open and closed arms of the elevated plus maze. (A), Animals pre-treated with vehicle (Veh) and then treated with progesterone (Prog) spent a significantly longer percentage of time in the open arms compared to all groups. This effect was blocked in the animals pre-treated with RU-486 (RU) and treated with progesterone. (B), Percent time spent in the closed arms of the elevated plus maze. Animals pre-treated with vehicle and then treated with progesterone spent a significantly shorter percentage of time in the closed arms of the maze compared to all other groups. (N = 7–9 animals per group; Bars indicate mean±SEM; * indicates a significant difference between animals treated with vehicle and progesterone and all other groups P<0.05).

As expected, P treatment reduced the anxiety-like behavior of male rats in the elevated plus maze (p = 0.019; Fig. 1a). Post hoc analysis further revealed that the group of animals that were pretreated with vehicle followed by P spent a larger percent of time exploring the open arms of the maze. There were no differences in percent open arm exploration in any of the other groups. In addition, as might be expected, the percent of time spent exploring the closed arms was decreased (p = 0.001; Fig. 1b) in males pre-treated with vehicle followed by P compared to all other groups. There were no differences in percent closed arm exploration in any of the other groups. More importantly, however, all of the anxiolytic-like actions of P in male rats were blocked by prior treatment with the PR antagonist, RU-486 (Fig. 1a and b). There was no difference in the total number of arm entries in each of the groups (p = 0.06 Table 1).

**Table 1:** Data are separated by task.

Task	Variable	V-O	V-P	RU-P	RU-O
**Light/dark chamber**	Latency to enter the dark chamber (sec)	15.94±4.49	26.09±2.72*	13.94±2.42	11.45±1.87
**Elevated Plus Maze**	# Total Entries	3.12±.51	4.75±.81	6.0±.94	3.8±.61
	% Time Open	6.8±3.10	42.33±15.89*	16.19±4.9	3.40±1.9
	%Time Closed	77.91±11.15	25.99±14.52*	72.68±9.47	84.10±7.19

Light/dark chamber; animals pre-treated with vehicle (Veh) and then treated with progesterone (Prog) had the longest latency to enter the dark side of the chamber. Elevated plus maze; there was no difference in total number of entries in any of the groups. (N = 6–9 animals per group; Data shown are mean±SEM; *indicates a significant difference between Veh-Prog and all other groups (p<0.01).

* indicates a significant difference between animals treated with vehicle and progesterone (V-P) and all other groups (p<0.01). N = 6–9 animals per group. V-O, Vehicle-Oil; RU-P, RU-486-Progesterone; RU-O, RU-486-Oil.

### The effect of RU-486 on the anxiolytic-like effect of P in the light/dark chamber

**Figure 2:**
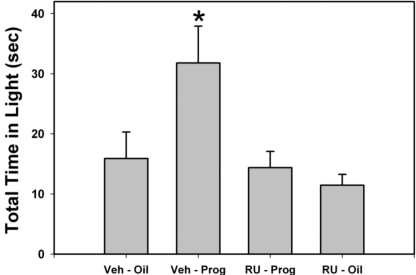
The effect of P and RU-486 on time spent in the light side of a Light/Dark chamber. Animals pre-treated with vehicle (Veh) and then treated with progesterone (Prog) spent a significantly longer amount of time on the light side of the chamber than all other groups. This effect was blocked by pre-treatment with RU-486 (RU). (N = 6–7 animals per group; Bars indicate mean±SEM; * indicates a significant difference between animals treated with vehicle and progesterone and all other groups (p = 0.006).

There was a significant effect of treatment on time spent on and latency to enter the light side of the light/dark box (p = 0.006; Fig. 2; p = 0.01; Table 1, respectively). Vehicle plus P treated animals spent more time on the light side of the chamber than males in any of the other treatment groups. There were no differences in time spent on the light side of the chamber in any of the other groups. In addition, animals pre-treated with vehicle followed by P also had longer latencies to cross over to the dark side of the chamber compared with all the other groups tested. There were no differences in latency to cross over to the dark side in any of the other groups. More importantly, post hoc comparisons indicate that the anxiolytic-like actions of P in male rats were blocked by prior treatment with the PR antagonist, RU-486 (Fig. 2 and Table 1).

### Hormone levels

**Figure 3:**
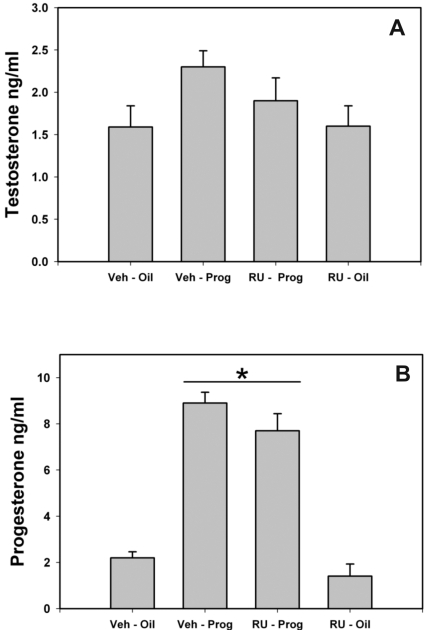
The effect of P and RU-486 on plasma testosterone and progesterone levels in male rats. (A), Testosterone levels were statistically the same in each treatment group. All animals in this experiment were castrated and implanted with testosterone-filled capsules. (B), Progesterone levels were higher in both groups of progesterone-treated animals compared to control groups. (Bars indicate mean±SEM; * indicates a significant difference between groups (p = 0.001).

The levels of T assayed in our animals was in the range of 1.5–2.5 ng/ml. This level is within the normal range for adult male rats [34]. The levels of T found in each of the treatment groups was similar (p = 0.194; Fig. 3a).

The levels of P was significantly increased by P treatment (p = 0.001; Fig. 3b). The animals not treated with P, the vehicle-oil and the RU-486-oil groups, had levels of P that were in the range of typical levels for males of this age [30], [31]. The level of P in the vehicle-oil and in the RU-486-oil groups was 2.2±.2 ng/ml and 1.4±.2 and 2.29±.26 ng/ml, respectively. The animals treated with P had much higher levels of P; however, these levels were similar to the physiological levels observed in animals that are exposed to stress paradigms [30]. The level of P in the vehicle-P and in the RU-486-P groups was 8.9±.4 and 7.7±0.7 ng/ml, respectively.

## Discussion

We report that P can have anxiolytic-like actions on behavior in male rats in two widely accepted tests of anxiety. Also, these actions can be virtually eliminated by blocking PR. Our results indicate that treatment with P decreases open arm avoidance on the elevated plus maze and increases the amount of time spent on the light side of a light/dark chamber. Importantly, prior treatment with the PR antagonist RU-486 blocks this anxiolytic-like behavior. We do not believe our effects can be attributed to antagonism of the glucocorticoid receptor, as corticosterone has the opposite effects on anxiety behavior as P [35]. We also report that P levels in animals that were treated with P were, as might be expected, significantly higher than the levels observed in our non-P treated control groups and that T levels are unaffected by treatment. Our behavioral results suggest that P acts on PR to reduce anxiety-related behavior in male rats. While both of our P-treated groups had physiological relevant levels of circulating P, the anxiolytic actions of P was blocked in those males pretreated with the PR antagonist RU-486. If reductions in anxiety-like behavior were solely a result of P metabolites acting on GABA_A_ receptors, both groups treated with P regardless of their pretreatment would still have shown a reduction in anxiety-like behavior, however they did not. Our data are also in agreement with data which have shown that P can function to influence a single behavior by activating both PR dependent and GABA receptor mediated pathways [17], [36]–[39].

PR dependent, or genomic, versus GABA receptor mediated, or non-genomic, mechanisms for steroid hormone action have been reported in modulating lordosis behavior, a measure of sexual receptivity, in female hamsters [36]. Sexual receptivity in female rodents is under the genomic control of systemic administration of sequential estrogen (E) and P [40]. Also, sexual receptivity can be facilitated non-genomically by GABAergic drugs implanted locally into specific brain areas [41]. Data from a series of experiments that examined genomic versus non-genomic regulation of lordosis behavior, suggest that P implanted into the ventromedial hypothalamus (VMH), and either P or its neuroactive metabolites implanted into the ventral tegmental area (VTA) facilitate lordosis [42], [43]. In the PR rich VMH, P functions in a genomic manner; in the VTA where PR are not as plentiful, the metabolites of P likely function in a membrane-mediated non-genomic manner. Based on these data and the data from the experiment presented here, we hypothesize that P may regulate anxiolytic-like behavior in male rats in a manner that is consistent with a dual pathway. We suggest that in one pathway, P influences anxiety following its metabolism to neuroactive steroids and subsequent modulation of the GABA_A_ receptor, indeed there is an overwhelming amount of evidence for this mechanism [44]. An additional, perhaps complementary, pathway may also exist by which P influences anxiety by interacting with PR.

A previous study showed that the PR antagonist RU-486 given to female rats did not block the anxiolytic-like effects of P [16]. These data provided support for the hypothesis that P modulates GABA_A_ receptors following its conversion to neuroactive steroids. There are some differences between that study and ours that may help to explain the discrepancies between our findings and theirs. One difference may be that we used males in our studies and the previous experiment used females, and PR function may differ between the sexes. Also, both studies used doses of P and RU-486 that were similar, however, the females in the previous experiment were tested in the elevated plus maze 4 hours after 1 P treatment. In the current experiment, we treated animals with P and/or RU-486 for three days. P can rapidly, in a non-genomic manner, influence anxiety via GABA_A_ receptors [10], perhaps then over a longer period of time, P can influence anxiety through mechanisms that require PR. While the exact time course for genomic versus non-genomic action to occur is unclear, it is often agreed upon that genomic action can be measured in hours or days while non-genomic action can take place in seconds and/or minutes [45], [46]. Indeed, we have shown that systems regulated by P are only influenced by P after multiple days (3–5) of treatment [47]. Based on this reasoning, it may be the time course of treatment that holds the answer to the differential results presented in the past and the data we present here.

A potential mechanism by which P may be acting on other systems in the brain may be through modulation of the vasopressin system. A role for altered transmission in the vasopressin system in mediating anxiety-related behaviors has been supported in numerous studies [48]–[51]. We have recently shown that treatment of male rats with P reduces the expression of vasopressin protein in portions of the vasopressin system [52]. These portions of the vasopressin system are highly sexually dimorphic (i.e., males have 5× as many cells and fibers in these areas as females), and steroid responsive [53], [54]. As P treatment can reduce vasopressin expression in the male brain, and reduced vasopressin action in the brain results in decreased anxiety-like behavior, the effect of P on anxiety-related behavior in male rats may be through modulation of the vasopressin system.

The distribution of PR in brain regions that are involved in anxiety is consistent with the idea that P may regulate anxiety by binding to intracellular PR. Indeed, the distribution of PR in both the male and female rat brain has been well described. A number of techniques, from binding assay to PCR, have been used to localize PR in the brain [47], [55]–[58]. Areas classically associated with reproduction, the preoptic area, the ventromedial, dorsomedial and arcuate nucleus of the hypothalamus all contain PR. In addition, PR has been reported in the olfactory bulb, frontal cortex, hippocampus, cerebellum and brainstem. Areas that have been implicated in the neural basis of fear, stress, and anxiety [59] also contain PR [47]. For example, the bed nucleus of the stria terminalis contains especially high numbers of PR and the amygdala, as well, contains PR immunoreactivity. These data suggest that P could act on PR in areas that play a large role in modulating anxiety-like responses.

The data presented here add to the growing body of knowledge as to the mechanisms by which P influences anxiety. Much of the data concerning the effect of P on anxiety have shown that P's effects are highly dependent upon dose. It is important to note that many of the studies investigating the anxiolytic actions of P use supraphysiological doses. In most of these studies, when dose is adjusted for species and size of animal, single doses range from about 5 to 50 times higher [1], [10], [18], [60] than the treatment course of P used in the current study. We chose to keep the current dose low, as male rats tend to have an average level of about 1.5–2.0 ng/ml circulating P [30], [31]. The dose of P used in our study results in physiological levels of serum P levels in males that resemble those of males enduring stress [30]. It is important to note, however, that the elevated levels of P seen in our animals were a result of 3 days of P treatment; a longer amount of time than elevated P levels seen in stress. Higher endogenous levels of P are coincident with higher levels of neurosteroids in the brain and in the circulation [4], and it therefore stands to reason that treatment with high doses of P result in higher levels of P metabolites. These higher levels of metabolites may have a more potent effect at GABA_A_ receptors. Indeed, dose response studies have shown that larger doses of P metabolites can have increasing anxiolytic-like effects [1], [15]. Taken together, it is conceivable that our animals may have had low levels of brain neurosteroids following treatment with low P doses, as is evidenced by physiological circulating levels of P.

In summary, our data suggest an additional pathway by which P can regulate anxiety-like behavior in males. They also support a physiologically and behaviorally relevant role for P in the male brain. While the data indicating a role for neuroactive steroids in the reduction of anxiety-like behavior are quite compelling and by no means in question, the data presented here suggest that there may be an important role for PR in the reduction of anxiety-like behavior in male rats.

## References

[1] Picazo O, Fernandez-Guasti A (1995). Anti-anxiety effects of progesterone and some of its reduced metabolites: an evaluation using the burying behavior test.. Brain Res.

[2] Bitran D, Hilvers RJ, Kellogg CK (1991). Anxiolytic effects of 3 alpha-hydroxy-5 alpha[beta]-pregnan-20-one: endogenous metabolites of progesterone that are active at the GABAA receptor.. Brain Res.

[3] Mora S, Dussaubat N, Diaz-Veliz G (1996). Effects of the estrous cycle and ovarian hormones on behavioral indices of anxiety in female rats.. Psychoneuroendocrinology.

[4] Frye CA, Petralia SM, Rhodes ME (2000). Estrous cycle and sex differences in performance on anxiety tasks coincide with increases in hippocampal progesterone and 3alpha,5alpha-THP.. Pharmacol Biochem Behav.

[5] Rodriguez-Sierra JF, Howard JL, Pollard GT, Hendricks SE (1984). Effect of ovarian hormones on conflict behavior.. Psychoneuroendocrinology.

[6] Toufexis DJ, Davis C, Hammond A, Davis M (2004). Progesterone attenuates corticotropin-releasing factor-enhanced but not fear-potentiated startle via the activity of its neuroactive metabolite, allopregnanolone.. J Neurosci.

[7] Tsai MJ, O'Malley BW (1994). Molecular mechanisms of action of steroid/thyroid receptor superfamily members.. Annu Rev Biochem.

[8] Mani SK, Blaustein JD, O'Malley BW (1997). Progesterone receptor function from a behavioral perspective.. Horm Behav.

[9] Mulac-Jericevic B, Conneely OM (2004). Reproductive tissue selective actions of progesterone receptors.. Reproduction.

[10] Gomez C, Saldivar-Gonzalez A, Delgado G, Rodriguez R (2002). Rapid anxiolytic activity of progesterone and pregnanolone in male rats.. Pharmacol Biochem Behav.

[11] Mellon SH, Griffin LD (2002). Neurosteroids: biochemistry and clinical significance.. Trends Endocrinol Metab.

[12] Robel P, Schumacher M, Baulieu EE, Baulieu EE, Robel P, Schumacher M (1999). Neurosteroids: From Definition and Biochemistry to Physiopathologic Function.. Neurosteroids: A new regulatory function in the nervous system.

[13] Paul SM, Purdy RH (1992). Neuroactive steroids.. FASEB J.

[14] Rupprecht R (2003). Neuroactive steroids: mechanisms of action and neuropsychopharmacological properties.. Psychoneuroendocrinology.

[15] Wieland S, Lan NC, Mirasedeghi S, Gee KW (1991). Anxiolytic activity of the progesterone metabolite 5 alpha-pregnan-3 alpha-o1-20-one.. Brain Res.

[16] Bitran D, Shiekh M, McLeod M (1995). Anxiolytic effect of progesterone is mediated by the neurosteroid allopregnanolone at brain GABAA receptors.. J Neuroendocrinol.

[17] McCarthy MM, Felzenberg E, Robbins A, Pfaff DW, Schwartz-Giblin S (1995). Infusions of diazepam and allopregnanolone into the midbrain central gray facilitate open-field behavior and sexual receptivity in female rats.. Horm Behav.

[18] Reddy DS, O'Malley BW, Rogawski MA (2005). Anxiolytic activity of progesterone in progesterone receptor knockout mice.. Neuropharmacology.

[19] Schneider JS, Burgess C, Sleiter NC, Doncarlos LL, Lydon JP (2005). Enhanced sexual behaviors and androgen receptor immunoreactivity in the male progesterone receptor knockout mouse.. Endocrinology.

[20] Frye CA, Walf AA, Rhodes ME, Harney JP (2004). Progesterone enhances motor, anxiolytic, analgesic, and antidepressive behavior of wild-type mice, but not those deficient in type 1 5 alpha-reductase.. Brain Res.

[21] Kellogg CK, Barrett KA (1999). Reduced progesterone metabolites are not critical for plus-maze performance of lactating female rats.. Pharmacol Biochem Behav.

[22] Pellow S, Chopin P, File SE, Briley M (1985). Validation of open∶closed arm entries in an elevated plus-maze as a measure of anxiety in the rat.. J Neurosci Methods.

[23] Walf AA, Frye CA (2007). The use of the elevated plus maze as an assay of anxiety-related behavior in rodents.. Nat Protoc.

[24] Gordon GG, Southren AL, Tochimoto S, Olivo J, Altman K (1970). Effect of medroxyprogesterone acetate (Provera) on the metabolism and biological activity of testosterone.. J Clin Endocrinol Metab.

[25] Satyaswaroop PG, Gurpide E (1978). A direct effect of medroxyprogesterone acetate on 17 beta-hydroxysteroid dehydrogenase in adult rat testis.. Endocrinology.

[26] Barbieri RL, Ryan KJ (1980). Direct effects of medroxyprogesterone acetate (MPA) and megestrol acetate (MGA) on rat testicular steroidogenesis.. Acta Endocrinol (Copenh).

[27] Zumpe D, Michael RP (1988). Effects of medroxyprogesterone acetate on plasma testosterone and sexual behavior in male cynomolgus monkeys (Macaca fascicularis).. Physiol Behav.

[28] Brown TJ, Blaustein JD (1984). Inhibition of sexual behavior in female guinea pigs by a progestin receptor antagonist.. Brain Res.

[29] Brown TJ, Blaustein JD (1986). Abbreviation of the period of sexual behavior in female guinea pigs by the progesterone antagonist RU 486.. Brain Res.

[30] Andersen ML, Bignotto M, Machado RB, Tufik S (2004). Different stress modalities result in distinct steroid hormone responses by male rats.. Braz J Med Biol Res.

[31] Auger CJ, Jessen HJ, Auger AP (2006). Microarray profiling of gene expression patterns in adult male rat brain following acute progesterone treatment.. Brain Res.

[32] Powers JB, Valenstein ES (1972). Individual differences in sexual responsiveness to estrogen and progesterone in ovariectomized rats.. Physiol Behav.

[33] Blaustein JD, Wade GN (1977). Sequential inhibition of sexual behavior by progesterone in female rats: comparison with a synthetic antiestrogen.. J Comp Physiol Psychol.

[34] Kalra PS, Kalra SP (1977). Circadian periodicities of serum androgens, progesterone, gonadotropins and luteinizing hormone-releasing hormone in male rats: the effects of hypothalamic deafferentation, castration and adrenalectomy.. Endocrinology.

[35] Korte SM (2001). Corticosteroids in relation to fear, anxiety and psychopathology.. Neurosci Biobehav Rev.

[36] DeBold JF, Frye CA (1994). Progesterone and the neural mechanisms of hamster sexual behavior.. Psychoneuroendocrinology.

[37] DeBold JF, Frye CA (1994). Genomic and non-genomic actions of progesterone in the control of female hamster sexual behavior.. Horm Behav.

[38] Cornil CA, Ball GF, Balthazart J (2006). Functional significance of the rapid regulation of brain estrogen action: where do the estrogens come from?. Brain Res.

[39] McEwen BS (1991). Non-genomic and genomic effects of steroids on neural activity.. Trends Pharmacol Sci.

[40] Powers JB (1970). Hormonal control of sexual receptivity during the estrous cycle of the rat.. Physiology and Behavior.

[41] McCarthy MM, Masters DB, Fiber JM, Lopez-Colome AM, Beyer C (1991). GABAergic control of receptivity in the female rat.. Neuroendocrinology.

[42] Pleim ET, Lisciotto CA, DeBold JF (1990). Facilitation of sexual receptivity in hamsters by simultaneous progesterone implants into the VMH and ventral mesencephalon.. Horm Behav.

[43] Frye CA, Leadbetter EA (1994). 5 alpha-reduced progesterone metabolites are essential in hamster VTA for sexual receptivity.. Life Sci.

[44] Dubrovsky B (2006). Neurosteroids, neuroactive steroids, and symptoms of affective disorders.. Pharmacol Biochem Behav.

[45] Rupprecht R, Holsboer F (1999). Neuropsychopharmacological properties of neuroactive steroids.. Steroids.

[46] McEwen BS, Coirini H, Schumacher M (1990). Steroid effects on neuronal activity: when is the genome involved?. Ciba Found Symp.

[47] Auger CJ, De Vries GJ (2002). Progestin receptor immunoreactivity within steroid-responsive vasopressin-immunoreactive cells in the male and female rat brain.. J Neuroendocrinol.

[48] Bielsky IF, Hu SB, Szegda KL, Westphal H, Young LJ (2004). Profound impairment in social recognition and reduction in anxiety-like behavior in vasopressin V1a receptor knockout mice.. Neuropsychopharmacology.

[49] Ring RH (2005). The central vasopressinergic system: examining the opportunities for psychiatric drug development.. Curr Pharm Des.

[50] Landgraf R, Gerstberger R, Montkowski A, Probst JC, Wotjak CT (1995). V1 vasopressin receptor antisense oligodeoxynuclotide into septum reduces vasopressin binding, social discrimination abilities, and anxiety-related behavior in rats.. J Neurosci.

[51] Caldwell HK, Lee HJ, Macbeth AH, Young WS (2008). Vasopressin: behavioral roles of an “original” neuropeptide.. Prog Neurobiol.

[52] Auger CJ, Vanzo RJ (2006). Progesterone treatment of adult male rats suppresses arginine vasopressin expression in the bed nucleus of the stria terminalis and the centromedial amygdala.. J Neuroendocrinol.

[53] De Vries GJ, Buijs RM (1983). The origin of the vasopressinergic and oxytocinergic innervation of the rat brain with special reference to the lateral septum.. Brain Res.

[54] De Vries GJ, Buijs RM, van Leeuwen FW, Caffe AR, Swaab DF (1985). The vasopressinergic innervation of the brain in normal and castrated rats.. J Comp Neurol.

[55] Sar M, Stumpf WE (1973). Neurons of the hypothalamus concentrate (3H)progesterone or its metabolites.. Science.

[56] Brinton RD, Thompson RF, Foy MR, Baudry M, Wang J (2008). Progesterone receptors: form and function in brain.. Front Neuroendocrinol.

[57] Rainbow TC, Parsons B, McEwen BS (1982). Sex differences in rat brain oestrogen and progestin receptors.. Nature.

[58] Blaustein JD, Wade GN (1978). Progestin binding by brain and pituitary cell nuclei and female rat sexual behavior.. Brain Res.

[59] Walker DL, Toufexis DJ, Davis M (2003). Role of the bed nucleus of the stria terminalis versus the amygdala in fear, stress, and anxiety.. Eur J Pharmacol.

[60] Frye CA, Sumida K, Dudek BC, Harney JP, Lydon JP (2006). Progesterone's effects to reduce anxiety behavior of aged mice do not require actions via intracellular progestin receptors.. Psychopharmacology (Berl).

